# Resilience as a Positive Youth Development Construct: A Conceptual Review

**DOI:** 10.1100/2012/390450

**Published:** 2012-05-02

**Authors:** Tak Yan Lee, Chau Kiu Cheung, Wai Man Kwong

**Affiliations:** Department of Applied Social Studies, College of Liberal Arts and Social Sciences, City University of Hong Kong, Hong Kong

## Abstract

The concept of resilience is reviewed from a range of disciplinary perspectives in this paper. Both broad and narrow definitions of resilience are highlighted and a working definition of resilience is proposed to inform research, policy and practice. Different psychological, social and ecological protective factors, particularly competence, optimism, and bonding to family and cultural beliefs are highlighted. Theoretical relationships between resilience and positive youth development are examined with an attempt to erase misunderstandings. Finally, how schools can promote resilience among students is discussed.

## 1. Background

Research on resilience has been a major theme in developmental psychopathology focusing on the question why some children and adolescents maintain positive adaptation [1] despite experiences of “distressing life conditions and demanding societal conditions” (p.1) [2] such as violence, poverty, stress, trauma, deprivation, and oppression. Despite concerted efforts in research on the concept of resilience over three decades, there are still different definitions of the term. Three waves of research on resilience have been identified and have set the path for the fourth wave which focuses on multilevel analysis and the dynamics of adaptation and change [3, 4]. Although resilience has been linked with positive youth development [5], there is a wide range of theories about the relationships between resilience and positive youth development. In order to promote an integration of theory, research, practice, and policy on positive youth development, a critical review of resilience is imperative. Consistent with the framework of applied developmental science, we offer a critical examination of several theories. In particular, the present paper reviews the theoretical conception of resilience, its relationships with positive youth development, as well as the antecedents of resilience. In addition, ways of enhancing adolescents' resilience that are pertinent to positive development are outlined.

## 2. Definitions of Resilience

In studying resilience, there are three critical conditions: (i) growing up in distressing life conditions and demanding societal conditions that are considered significant threats or severe adversities, (ii) the availability of protective factors, including internal assets and external resources that may be associated with counteracting the effects of risk factors, and (iii) the achievement of positive adaptation despite experiences of significant adversity [4, 6–11].

 A *broad* definition was given by Masten and colleagues [8] defining resilience as the process of, capacity for, or outcome of successful adaptation despite challenging or threatening circumstances. Since then, difficulties in defining resilience have become more widely recognized [3, 4, 12–14]. In explaining why some children and adolescents maintain positive adaptation even though they grow up in deprived, troubled, and threatening environments, differences in measuring the significance, quality, and quantity of adversities as well as positive adjustment are commonly found. The American Psychological Association also uses a broad definition: “the process of adapting well in the face of adversity, trauma, tragedy, threats, or even significant sources of stress—such as family and relationship problems, serious health problems, or workplace and financial stressors. It means “bouncing back” from difficult experiences” [15]. However, a review of existing studies indicates that the proportions of “resilient” youth varied from 25 to 84% [16]. This finding supports the adoption of a *narrow* definition of resilience focusing on specific development outcomes at different specific points in life [16]. 

 Benson [17] postulated that the term “resilience” indicates a paradigm shift from the identification of the risk factors of an individual (i.e., a pathological view) to the identification of strengths of an individual. A “resilient” individual is stress-resistant and less vulnerable despite experiences of significant adversity [18].

 To sum up, resilience can be defined in terms of an individual's capacity, the process he or she goes through, and the result [8]. Resilience as a *capacity* refers to an individual's capacity for adapting to changes and stressful events in a healthy way [5]. Resilience as a *process* is regarded as a reintegration process and a return to normal functioning with the support of protective factors after encountering a severe stressor [19]. Resilience as a *result* is defined as the positive and beneficial outcomes resulting from successfully navigating stressful events [8]. Resilience has been defined as a multidimensional construct in its operational characteristics, and a key variable in predicting positive outcomes in the face of adversity. Therefore, an operational definition of resilience must encompass all of the key characteristics of resilience and include the components of capacity, process, and result. Therefore, resilience can be defined as the process of effectively mobilizing internal and external resources in adapting to or managing significant sources of stress or trauma. Thus, cultivation of resilience means fostering adolescents' capacity, flexibility, and coping strategies as they face developmental changes and life stresses in order to “bounce back” from difficult life experiences and achieve positive outcomes [5, 20, 21].

## 3. Four Waves of Research on Resilience

The complicated methodological issues of identifying antecedents, defining adversity, and specifying the consequences of resilience are not easy to resolve [4, 13]. This complexity creates considerable challenges for defining resilience. Furthermore, differences in definition lead to variations with regard to the nature of potential risk and protective processes [12–14]. However, researchers have found many correlates of resilience (protective factors) among studies that used varying measurement strategies. Also, a synthesis of methodological approaches can shed light on a clear identification of the antecedents, defining attributes and outcomes of resilience. For example, Masten and Obradović [3, 22] have summarized the first three waves of resilience research: (i) identifying the correlates and characteristics of good adaptation among children and adolescents who appear to develop well despite genetic or environmental risks, (ii) uncovering the processes and regulatory systems that explain how potential assets or protective factors work, and (iii) promoting resilience through prevention, intervention, and policy as a result of the concomitant rise of prevention science which emphasizes the importance of promoting competence as a strategy. These three waves of research contributed significantly in terms of concepts, methods, findings, issues, controversies, and clues that are useful in promoting a new wave of research.

 The latest wave of research adopts a systems perspective and makes use of advanced technologies of measurement and analysis of multiple levels of functioning. It also focuses on gene-environment interactions as well as the development of adaptive systems. In a review by Masten and Obradović [22], the following fundamental adaptive systems that play a crucial role in resilience have been identified: (i) learning systems of the human brain (problem-solving, information processing), (ii) attachment system (affective processes), (iii) mastery motivation system (self-efficacy processes), (iv) stress response systems (alarm and recovery processes), (v) self-regulation systems (emotion and behavior regulation), and other systems including family, school, peer, as well as cultural and societal systems. Among them, research on psychological stress and ways of coping with stress attracts a lot of attention because these factors are crucial in the models of resilience for children and adolescents [23–25]. Psychological and biological processes of reaction to and recovery from stress play a central role in understanding how prolonged exposure to chronic stress exacts physical and emotional tolls. In a review of the psychobiological processes of stress and coping, Compas [26] summarized substantial evidence suggesting that automatic responses to stress, including emotional and physiological arousal, impulsive action, intrusive thoughts, and some forms of escape behavior, may be activated by triggering the amygdala in response to threat in the environment. Researchers use advanced methods to examine the structure and function of the brain and central nervous system in order to illuminate the neurobiological structure and processes of human coping and adaptation to stress. Compas [26] pointed out that recent research findings also provide evidence to support that “coping is a part of the overall set of executive functions that are regulated by the prefrontal cortex” (p.230).

 In sum, the latest developments clearly point to an integration of biological, psychological, and social perspectives building on evidence gathered from the first three waves of resilience research.

## 4. Protective Factors for Psychosocial Resilience in Children and Adolescents

Studies have shown that the main difference between individuals who adapt very well despite facing risks and individuals who end up in maladaptation is the existence of protective factors. Thus, enhancing both internal and external protective factors of adolescents may help them adapt to stressful and risky life situations. For *internal protective factors*, Smith [27] summarized research findings and found that optimism, perceptions of control, self-efficacy, and active coping are associated with better health. Grotberg [28] cited longitudinal studies to show that about half to two-thirds of children with resilience could overcome their initial traumatic life experiences, such as growing up in families with a mentally ill member, being abused, or having criminally involved parents. Thus, cultivating resilience is an important way to promote the psychological and social development of adolescents. For *external protective factors*, theorists [29] have suggested that people who do not have a functional social support system are more vulnerable to external stresses. Therefore, it is important to strengthen an individual's ability to recognize and utilize social support systems in his or her surroundings. There is a growing consensus from child and adolescent research on important protective factors [3, 30]. They can be summarized and grouped into four main components as follows. However, the salience of these factors may vary across the life span.

**Bonding**

It consists in emotional attachment and commitment to parents or caregivers (particularly those who maintain a positive family climate, experience a low level of conflict, and are involved in the child's education), close relationships with mature and supportive adults, connections to prosocial and rule-abiding friends, and bonding to people in prosocial organizations.

**Competence**

Five core individual competencies are involved. (i) cognitive competence, that is, good cognitive abilities, (ii) emotional competence in terms of good self-regulation of emotions and impulses, (iii) moral competence, that is, positive self-perceptions, (iv) behavioral competence, that is, talents valued by self and society, and (v) social competence, that is, general appeal or attractiveness to others.

**Optimism**

It is manifested self-efficacy, spirituality, that is, faith and a sense of meaning in life, as well as a clear and positive identity.

**Enviroment**

For example, organized home environment, authoritative parenting (high on warmth, structure/monitoring, and expectations), socioeconomic advantages, effective schools, neighborhoods with high “collective efficacy,” high level of public safety, good emergency social services, as well as good public health and health care availability.

The above list is not exhaustive. A growing body of literature supports the notion that resilience can also be enhanced by an ethnic family's cultural values and provision of mutual psychological support [31–33]. Furthermore, some internal assets may require two or more of the above protective factors. For example, research findings have suggested that a sense of humor, combining cognitive competence with an optimistic outlook, is an internal protective factor that alleviates an individual's focus on personal failure [34, 35]. Humor is therapeutic for managing anxiety and creates a buffer for individual against the negative effects of stress [36]. A good sense of humor is also positively related to a healthy self-concept [37]. Dixon [38] also pointed out that humor helps restructuring the cognitive perception of the threatening situation. Thus, it allows the adolescent to explore cognitive alternatives and develop conflict management strategies in response to stressful and threatening situations. It is expected that these skills are better managed by adolescents who have gained certain social and cognitive competencies [39].

## 5. Theoretical Relationships between Resilience and Positive Youth Development

Resilience researchers have conceptualized the relationship between adversity and competence differently [13], and these different conceptual models have led to differing analytic strategies. Some have used person-based data analytic approaches, which involve identifying individuals with high risk and high competence, and comparing them with low risk and high competence. Others have used variable-based analyses and found either main effects or interaction effects. This diversity in analytic structure and measurement reflects the need for both a clarification of different definitions of resilience and a critical examination of the conceptualized relationships between resilience and positive youth development.

 According to various theories or models, there are eight possible relationships between resilience and positive youth development. Four of the relationships take resilience as a forerunner of positive youth development, and four others regard resilience as a result of positive youth development. The distinction between the forerunner and the result represents a dimension of role. Alternatively, the eight relationships reflect four modes of conditionality, pertaining to the sufficient, necessary, probabilistic, and spurious conditions [40]. A sufficient condition is able to invoke something solely. In contrast, a necessary condition is something must be present. A probabilistic condition is likely to invoke something, usually contingent on other conditions. This represents neither a sufficient nor a necessary condition. A spurious condition does not invoke something and may merely represent coincidence. Combining the dimensions of role and conditionality thereby identifies eight possible relationships such that resilience is a (1) constituent, (2) determinant, (3) contributor, (4) concomitant, (5) indicator, (6) a derivative of positive youth development, (7) a collateral of positive youth development due to a common effect, and (8) collateral of positive youth development due to a common cause (see Table 1).

Resilience as a *constituent* maintains that it is a sufficient forerunner to define positive youth development. As such, resilience is a defining condition for positive youth development and alternatively positive youth development must follow resilience. This is the view of the asset-building model and the inclusiveness model of positive youth development. Firstly, the asset-building model posits that resilience is one of the youth's internal assets for constituting positive youth development and as such, the development refers to the process of asset building [41]. In this connection, resilience would have an association with similar assets such as the optimism, controllability, conflict resolution, and problem-solving aspects of positive youth development [42, 43]. In this model, all these assets are constituent or sufficient conditions to positive youth development. Moreover, positive youth development also hinges on external assets. A notable instance of asset building happens in the caring school, which provides opportunities or challenges for realizing resilience [44]. Secondly, the inclusiveness model, which incorporates the asset building approach, holds that resilience is particularly a constituent of positive youth development in an inclusive or comprehensive way [45]. As such, the inclusiveness model regards resilience as the key to relationship building and engagement of social support, which defines the inclusiveness required for positive youth development. Essentially, the inclusiveness model states that personal strengths such as resilience is a constituent of social inclusiveness and this inclusiveness is then a component of positive youth development. Both the asset-building model and the inclusiveness model thereby define positive youth development in terms of the use of strengths or assets such as resilience in the developmental process. Notably, positive youth development in this case refers to the process of asset building and inclusiveness. It is, therefore, an emergent or induced variable contingent on resilience [46, 47]. Essentially, resilience constitutes asset building and inclusiveness, which are tantamount to positive youth development according to the models.

Resilience as a *determinant* or strong predictor means that it is a necessary forerunner giving rising to positive youth development. As a necessary forerunner, resilience is not something to define positive youth development. Instead, resilience only functions as a very important predictor of positive youth development. Hence, resilience and positive youth development can be separated such that the former does not necessarily create the latter. Despite that, positive youth development would be a distinctive outcome highly dependent on resilience. This is the view of both the courage model of resilience and the problem avoidance model of positive youth development. The courage model maintains that resilience embodies courage for positive youth development through the manifestations of belonging, mastery, independence, and generosity. These characteristics then satisfy needs for attachment, achievement, autonomy, and altruism [48]. Therefore, resilience represents a mental force to engender positive youth development through need fulfillment. The problem-avoidance model, alternatively, posits that resilience is a necessary condition for positive youth development [45]. As such, positive youth development is only possible in the absence of problems, as problems are usually impediments to learning and growth. Essentially, this model contrasts with the inclusiveness model, which regards resilience as a sufficient condition for positive youth development.

Resilience as a *contributor* to or probabilistic condition for positive youth development means that it is likely to induce the development or resilience, but the likelihood is neither compelling nor straightforward. This role of resilience is inherent in the developmental systems theory of positive youth development [49, 50]. This theory maintains that positive youth development results from the alignment of personal strengths and community assets. As such, the function of resilience as a personal strength is contingent on the support and opportunities available in the context and the program. When the context or program encourages or requires resilience, resilience would become a determinant of positive youth development. The theory also posits the presence of multiple systems, each of which interactively contributes to positive youth development. Therefore, the personal strength of resilience is one factor, playing the role of a contributor, collaborating with other factors in the production of positive youth development.

Resilience as a *concomitant* that follows positive youth development means that positive youth development is a sufficient condition for resilience. That is, positive youth development alone is capable of generating resilience. This is the view of the solution-focused model of resilience, which regards resilience as success in development, adaptation, or overcoming problems, or simply as a solution to problems [51]. In this view, positive youth development means resilience, as a result of successfully encountering developmental tasks or problems [52–56]. In other words, because of difficulties in development, resilience takes shape in the success of positive youth development or in solutions to developmental problems. Resilience is, therefore, not separate from positive youth development. Possibly, positive youth development is a process that results in the development of resilience.

Resilience as an *indicator* of positive youth development means that positive youth development is a necessary condition for resilience, and resilience necessarily reflects positive youth development. This is the view of the adaptation and competence models of positive youth development. The adaptation model holds that adaptation to myriad developmental tasks is imperative for positive youth development and the adaptation generates competence which upholds resilience [57]. Such competence comprises abilities to maintain a positive self-image, self-control, decision-making, moral reasoning, and social connectedness. Similarly, the competence model includes resilience as one among many forms of competence, including social competence, emotional competence, moral competence, self-determination, spirituality, and belief in the future. Together the development of these characteristics are indicative of positive youth development [5]. In this model, positive youth development is a latent variable, which is identifiable by resilience and other forms of competence.

Resilience as a *derivative* or probabilistic consequence of positive youth development means that human development is likely to engender resilience. This implies that resilience and positive youth development are conceptually separate and related only contingently. This implication inheres in self-regulation theory, which posits that positive youth development generates resilience in the presence of problems and alternative goal evaluations [58]. Self-regulation theory essentially holds that proactive action and expectation play a contributory role in tackling contextual problems. Relevant to positive youth development are selection, optimization, and compensation in the presence of problems [49, 59]. Accordingly, problems limit choices such that the selection of options for their best use and disallowing forbidden options is necessary. Self-regulation demonstrates its usefulness in tackling problems, creating the need for change or self-regulation. Key to the probabilistic influence of positive youth development is confidence, which indicates thriving or flourishing [49, 50, 60, 61].

Resilience holds a spurious relationship with positive youth development because their *common effect* means that the common effect is responsible for maintaining a relationship that otherwise does not hold. This is possible based on the citizenship model, which posits that both resilience and positive youth development are contributors to citizenship in terms of personal and social responsibility [49, 62–64]. Hence, both resilience and positive youth development serve a similar role in satisfying societal needs [63]. This similarity forms a relationship between resilience and positive youth development because of their common role.

Resilience has a spurious relationship with positive youth development due to their *common cause* means that the common cause implies a relationship that would not otherwise exist. This common causation is proposed in control theory, which posits that control is a common cause of both resilience and positive youth development [65]. Accordingly, control involves primary and secondary forms of control dealing with selection and compensation of factors and resources used to facilitate resilience and positive youth development. All these factors lead to coping, which is then conducive to resilience and positive youth development [66–70]. Hence, control and/or coping are common causes of both resilience and positive youth development, thus creating an illusion of relationship.

## 6. Discussion

The aforementioned eight possible relationships between resilience and positive youth development are not necessarily mutually exclusive, since they can operate at the same time in an additive way. This is because both resilience and positive youth development can take many forms, as either dynamic processes or static conditions. Nevertheless, the most viable, suitable, reasonable, and popular possibility is that resilience is a contributor to positive youth development, as based on developmental systems theory. This conceptualization has the advantage of treating resilience and positive youth development as separate concepts, which avoids confusion and overlap. The separation is vital for establishing discriminant validity and thereby the unique value of the two concepts. In this conceptualization, positive youth development has its own indicators. Consistent with developmental systems theory, the indicators are the six Cs of confidence, competence, connection, character, caring, and contribution [49, 50, 60, 61]. They make positive youth development conceptually different from resilience. Moreover, the contributory relationship does not require either a sufficient or a necessary condition in the relationship between resilience and positive youth development. This condition is easily and commonly met in empirical research [60, 68]. Most importantly, this formulation has a strong theoretical base in developmental systems theory [49, 50]. The theory tends to be realistic in regarding youth development as a product of interactions among multiple systems. Another strong justification is the differentiation of views that resilience deals with the removal of negative development problems and that positive youth development is about the positive side of development beyond problem resolution [13, 71–75]. Accordingly, the removing of problems in resilience is unlikely to represent or create positive youth development immediately. Furthermore, a third forceful justification is that resilience contributes to positive youth development only conditionally, in the presence of adversity or problems [13, 75]. This view is also consistent with developmental systems theory, which envisions positive youth development as a contingent outcome resulting from interactions among systems.

 Evidence supporting the conditional or probabilistic contribution of resilience to positive youth development, including its five major indicators of competence, confidence, connectedness, character, and caring, includes the following. First, resilience in terms of controllability over stress appears be more conducive to youth development in relation to stress-related growth when the youth has practiced problem-focused coping strategies. This is evidenced by enhanced competence. Controllability itself has not shown a main effect [68]. This conditional contribution implies that stress or adversity is needed for coping, and that enhanced competence is the successful consequence. When coping and controllability fit the need for coping, youth development emerges. Second, resilience in terms of residential stability in a disadvantaged neighborhood has appeared to be particularly conducive to positive youth development in terms of competence [76]. In this case, the disadvantaged neighborhood would be a source of adversity, giving rise to the opportunity for resilience to manifest. Third, resilience in terms of the absence of social anxiety has appeared to be more conducive to positive youth development in terms of the character of moral behavior when the youth has had a chronic illness [77]. In this connection, chronic illness as adversity combined with resilience can lead to reduced social anxiety and improved character, another major indicator of positive youth development. Fourth, resilience in terms of belief in a just world has appeared to be particularly conducive to self-esteem development in terms of anger induction [78]. As such, anger induction is an adversity, and the resilient response leads to enhanced confidence. Fifth, resilience in terms of absence of worry about illness appears conducive to the childhood cancer survivor's confidence [79]. Sixth, resilience in terms of morale in the presence of illness has appeared to foster development in terms of social interaction and relationship quality, which are defining characteristics of connectedness [80]. The latter two findings consistently show that illness can be an adverse condition which, when responded to with resilience, provides an important developmental contribution.

 One of the factors that may hinder the development of resilience research is the complexity of adversity. Future theoretical development needs to clearly define adverse events in the external world. Within a life-span developmental perspective, the context of the adversity could be biological, psychological, economic, or social. A major concern is that it will be inappropriate to apply the concept of resilience if a stressor does not require adaptation or does not lead to negative outcomes [81]. Not all adversities are equivalent in severity [16]. Therefore, research methodologies should carefully consider the identification of the specific adversity along with its severity and duration when constructing measurement instruments.

## 7. Cultivating Adolescents' Resilience in Schools

There are several ways to foster students' resilience in schools. First, schools can arrange curricula-based programs [5, 82–84], since many of these programs have been evidenced to enhance students' bonding, core competencies, and optimism through which students build up resilience. Comprehensive programs, such as the Project P.A.T.H.S. [83, 84] cover not only resilience, but also bonding, five core individual competencies, that is, cognitive, emotional, moral, behavioral, and social competencies, self-efficacy, spirituality, and a clear and positive identity as crucial elements in building resilience. Moreover, these programs can incorporate positive social norms, cultural values and ideologies to cultivate adolescents' prosocial attitudes, and an optimistic outlook towards the future that are crucial for cultivating adolescents' resilience.

 Second, it has been found that attachment to adults other than a child's parents has positive effects on a child's resilience to adversity [11, 85]. Also, bonding to school teachers increases positive developmental outcomes [86]. Therefore, schools can develop a culture that promotes two primary and interdependent components of school bonding: (i) *attachment:* close affective relationships with teachers at school and (ii) *commitment*: an investment in school and doing well in school because students will acquire teachers' values through a socialization process. Subsequently, these values will serve as a mediator of the effect of bonding on behavioral outcomes [86].

 Third, extra-curricular activities can be used to facilitate and maintain the healthy development of adolescents, but the effectiveness of these activities depends on the type, frequency, and quality of interchanges in the activity context [87]. Besides, resilience-focused groups can be used for students who need more intensive intervention due to the severity of adversity [88]. In addition, specialized intervention programs such as adventure-based counseling can be used [21].

 Finally, school social workers can collaborate with students' parents to encourage parental involvement and support in fostering the development of adolescents' resilience. Since adverse events affect behaviors of family members in terms of family rules, organizational structures, communication patterns, and beliefs systems, the ability to survive and recover from disruptive family life challenges is related to the family relationship network [89]. In general, the school can adopt a whole-school approach to involve different stakeholders in the school, family, and community to nurture the development of adolescents' resilience.

## 8. Conclusion

This paper endeavors to clarify the range of possible relationships between resilience and positive youth development according to various theories or models and perspectives. Among the four possible relationships that treat resilience as a precursor, the developmental systems model is identified as a contributor that may be considered as a weak predictor. However, resilience research has now moved to the fourth wave, adopting neuroscientific and biological approaches to the study of resilience, and taking advantage of technological advancements in measurement and analysis at multiple levels of functioning, including gene-environment interactions and adaptive systems. Hence the developmental systems model has a promising future. From a developmental perspective, adolescents within their natural contexts need to be studied in tandem and over time. This requires more sophisticated research designs. With respect to the cultivation of resilience among adolescents, one implication is that as adolescents develop toward adulthood, adverse situations will change as will their need for competencies. Therefore, a good person-stage-environment fit is required to keep pace with these changing needs and situations so that intervention programs remain developmentally appropriate to the target population.

 Furthermore, protective factors operate across different levels. In order for research to be realistic and interventions to be effective, we must consider how individual capacity interplays with external protective factors. There is a need for more research on the interactions among adversities, internal and external protective factors, and interventions.

## Figures and Tables

**Table 1:** Models relating resilience and positive youth development.

Conditionality	Resilience as a forerunner	Resilience as a follower
Sufficient	*Constituent * Asset building model Inclusiveness model	*Concomitant * Solution-focused model
Necessary	*Determinant* or substantial predictor Courage model Problem avoidance model	*Indicator * Adaptation model Competence model
Probabilistic	*Contributor* or weak predictor Developmental systems model	*Derivative * Self-regulation theory
Spurious	*Collateral due to a common effect * Citizenship model	*Collateral due to a common cause * Control theory
